# Scaling up care for perinatal depression for improved maternal and infant health (SPECTRA): protocol of a hybrid implementation study of the impact of a cascade training of primary maternal care providers in Nigeria

**DOI:** 10.1186/s13033-021-00496-6

**Published:** 2021-09-20

**Authors:** Oye Gureje, Bibilola Oladeji, Olatunde Olayinka Ayinde, Lola Kola, Jibril Abdulmalik, Waheed Akinola Lanre Abass, Neda Faregh, Phyllis Zelkowitz

**Affiliations:** 1 Department of Psychiatry, University of Ibadan, Ibadan, Nigeria; 2 Oyo State Primary Health Care Board, Ministry of Health, Oyo State Secretariat, Ibadan, Nigeria; 3 Department of Psychology, Carleton University, Ottawa, ON Canada; 4 Division of Social and Transcultural Psychiatry, McGill University, Montreal, Canada; 5 Department of Psychiatry, Jewish General Hospital, Montreal, Canada

**Keywords:** Perinatal depression, Primary care, Implementation study, MhGAP-IG

## Abstract

**Background:**

The large treatment gap for mental disorders in low- and middle-income countries (LMIC) necessitates task-sharing approaches in scaling up care for mental disorders. Previous work have shown that primary health care workers (PHCW) can be trained to recognize and respond to common mental disorders but there are lingering questions around sustainable implementation and scale-up in real world settings.

**Method:**

This project is a hybrid implementation-effectiveness study guided by the Replicating Effective Programmes Framework. It will be conducted in four overlapping phases in maternal care clinics (MCC) in 11 local government areas in and around Ibadan metropolis, Nigeria. In Phase I, engagement meetings with relevant stake holders will be held. In phase II, the organizational and clinical profiles of MCC to deliver chronic depression care will be assessed, using interviews and a standardized assessment tool administered to staff and managers of the clinics. To ascertain the current level of care, 167 consecutive women presenting for antenatal care for the first time and who screened positive for depression will be recruited and followed up till 12 months post-partum. In phase III, we will design and implement a cascade training programme for PHCW, to equip them to identify and treat perinatal depression. In phase IV, a second cohort of 334 antenatal women will be recruited and followed up as in Phase I, to ascertain post-training level of care. The primary implementation outcome is change in the identification and treatment of perinatal depression by the PHCW while the primary effectiveness outcome is recovery from depression among the women at 6 months post-partum. A range of mixed-method approaches will be used to explore secondary implementation outcomes, including fidelity and acceptability. Secondary effectiveness outcomes are measures of disability and of infant outcomes.

**Discussion:**

This study represents an attempt to systematically assess and document an implementation strategy that could inform the scaling up of evidence based interventions for perinatal depression using the WHO mhGAP-IG in LMIC.

*Trial registration* This study was registered on 03 December, 2019. https://doi.org/10.1186/ISRCTN94230307.

## Background

It is now commonly accepted that the mental health treatment gap in low- and middle-income countries (LMIC) requires a shift in policy and health planning in which focused attention is given to the horizontal integration of mental health into primary and maternal health care. This is because not only is mental health care an integral component of holistic, person-centred maternal care, there are also inherent benefits to perinatal women when mental health is embedded within routine maternal care. Some of these benefits include early and increased detection of mental health conditions, improved accessibility to mental health care, reduced stigma, as well as an intimate link of mental health to the maternal care needs of perinatal women [1]. The case for the integration of mental health into routine maternal care is further strengthened by the fact that the material and human resources necessary to respond to the burden of mental disorders, including perinatal depression, are grossly inadequate in most LMIC. For example, Nigeria has about 250 practicing psychiatrists for a population of over 200 million people [2]. The situation is worse for other mental health specialists such as clinical psychologists and social workers. The few available specialists are mostly based in urban areas and are therefore inaccessible to the majority of the population who resides in rural settings. The integration of mental health into primary care requires that the providers at that level of care are empowered with the skills necessary for them to offer basic but essential service for common mental health problems. A recent situation analysis of maternal mental health in primary care in five LMIC (India, Nepal, Uganda, South Africa and Ethiopia) [3] found that while most of the countries had a national mental health policy that included maternal mental health, almost all of them did not have a national plan that included dedicated maternal mental health services. In all of the LMIC, perinatal women could only access mental health services through referral to mental health specialists at district or specialist centres, some of which were several kilometres away.

Perinatal depression occurs in up to 10% of women prenatally and 13% postnatally in high-income countries [4]. There is evidence suggesting higher rates in LMIC. In a recent systematic review, the weighted mean prevalence for common perinatal mental disorders was 16% in the antenatal period and 20% postnatally in LMIC [5]. Perinatal depression is associated with long-term adverse consequences for maternal wellbeing and infant development. Perinatal depression is associated with suffering and loss of productivity [6], and is an important risk factor for maternal suicide [7]. Child adverse consequences of perinatal depression include pre-term birth and low birth weight, poor mother–child interactions, infant under-nutrition and stunted growth, elevated rates of diarrhoeal diseases, poor infant development, insecure attachment and higher rates of emotional and behavioural problems in infants of depressed mothers [8]. There are indications that these adverse child outcomes are worse in LMIC [9]. A large cohort study of more than 20,000 perinatal women in Ghana found that antenatal depression was associated with prolonged labour, peripartum and postpartum complications, non-vaginal delivery and newborn illness [10]. There is evidence that effective and cost-effective treatments exist for this condition [11, 12] and that frontline primary care workers, including non-physician primary care providers and midwives, can deliver evidence-based intervention to affected mothers [13–15]. However, even in high income countries (HIC), only a minority of depressed persons get the care they need, with estimates suggesting that less than 50% of cases of postnatal depression are detected by primary health care professionals in routine clinical practice [16].

As part of efforts to facilitate task sharing and enable front-line providers deliver evidence based care for common mental health conditions, the World Health Organization, through a systematic, consultative and participatory process, developed the mental health Gap Action Programme Intervention Guide (mhGAP-IG) for use by non-specialists [17]. The mhGAP-IG presents an evidence-based framework for the management of priority disorders using protocols for clinical decision-making within routine clinical service in non-specialist settings. Depression, including that occurring in women in the perinatal period, is one of the priority disorders included in the mhGAP-IG.

In pilot exploratory studies, we have shown that it is feasible to train non-physician primary care workers in the use of mhGAP-IG to deliver care for a range of mental health conditions, including depression [18]. In a subsequent fully-powered randomized controlled trial, women with perinatal depression, other than those with severe form of the disorder, treated by frontline providers using the basic psychological approaches described in mhGAP-IG had similar rate of remission as those treated with more intensive psychological interventions [19]. In that trial, treatment of depression according to the mhGAP intervention guidelines constituted the “enhanced care as usual” (the comparison group). There was no “care as usual” group as a second comparison group. At 6 month follow-up, two thirds of the women in the enhanced care as usual had attained remission from depression, suggesting that the mhGAP-IG might be a useful tool for scaling up care for perinatal depression by non-specialist frontline providers working in low- and middle-income countries. The availability of evidence-based guidelines and approaches for managing perinatal depression [11, 12], with proven efficacy and cost-effectiveness, has opened up the possibility of scaling up service for this disabling condition. However, information is lacking as to how these guidelines and approaches can be delivered within an integrated routine perinatal care. There is a need to demonstrate the goodness of fit of these approaches to the extant health systems of LMIC, and that these approaches can be implemented in non-specialist, indeed non-physician, health care settings that characterize primary maternal care in most of sub-Saharan Africa. It will be important to empower community midwives and primary care providers with the skills to detect and respond to mental health conditions at the maternal primary care level. While the WHO has prepared, and made available several modules of training packages, there remains the need to find skilled trainers. In LMIC, where specialists are few and often overwhelmed by the demands of providing care for the teeming numbers of people in need, such specialists lack the time to provide the necessary training for the many end users (primary care workers) to improve service delivery.

In an earlier study, we have demonstrated the impact of a cascade training format for the mhGAP-IG, (where mental health specialists (designated as Master Trainers) train non-specialist physicians and senior nurses (Trainers) who in turn deliver training to other primary care providers) in increasing the recognition and care for mental disorders in primary care [20]. Over the course of 12 months post-training, there was an average of 400% increase in the proportion of patients attending the clinics who received a mental, neurological or substance abuse (MNS) diagnosis [20]. There is a need to provide more robust evidence for the utility of this approach for scaling up care for mental health in routine primary care in this and other resource constrained settings.

Specifically, there are outstanding issues that require attention. First, there is a need to more closely study factors that may facilitate or impede a sustainable approach to training primary care providers to use mhGAP-IG as a clinical support tool for the delivery of effective intervention for mental health conditions in routine practice at the primary care level. Second, it is important to demonstrate whether the skill acquired following training is retained beyond the immediate post-training period and what factors affect skills retention. Complementary to this is the need to evaluate what level of refresher training might be required to sustain the clinical competence of trained primary care workers. Third, the fidelity of use of mhGAP-IG specifications need to be determined as well as factors that might affect fidelity. Fourth, the effectiveness and cost-effectiveness of recognition and treatment of mental disorders by the trained providers need to be demonstrated.

## Methods and design

### Objectives

The overall aim of the programme of work is to study factors that may impede or facilitate the delivery of evidence-based intervention for perinatal depression by front-line clinicians using the mhGAP-IG in routine practice. The knowledge so gained, including that gained in the process of responding to barriers that may be encountered, will provide necessary information to facilitate the scaling up of the intervention in resource-constrained settings.

The specific objectives are to:

1. Identify optimal organizational and health system-level processes for the effective implementation of mhGAP-IG in routine maternal care at the primary care level.
2. Implement a training design that prepares primary care providers to deliver effective and evidence-based intervention for perinatal depression in a sustainable way and that builds a pool of trainers within the health system;
3. Assess the effectiveness of the intervention on maternal and infant outcomes;
4. Determine the barriers and facilitators of scaling up the intervention into routine perinatal care; and
5. Provide population-level estimates of the cost and impact of scaling-up care for perinatal depression.

### Design

This hybrid (implementation-effectiveness) study will use a mixed-methods design and adopt a participatory research approach in all the stages of its implementation.

**Fig. 1:**
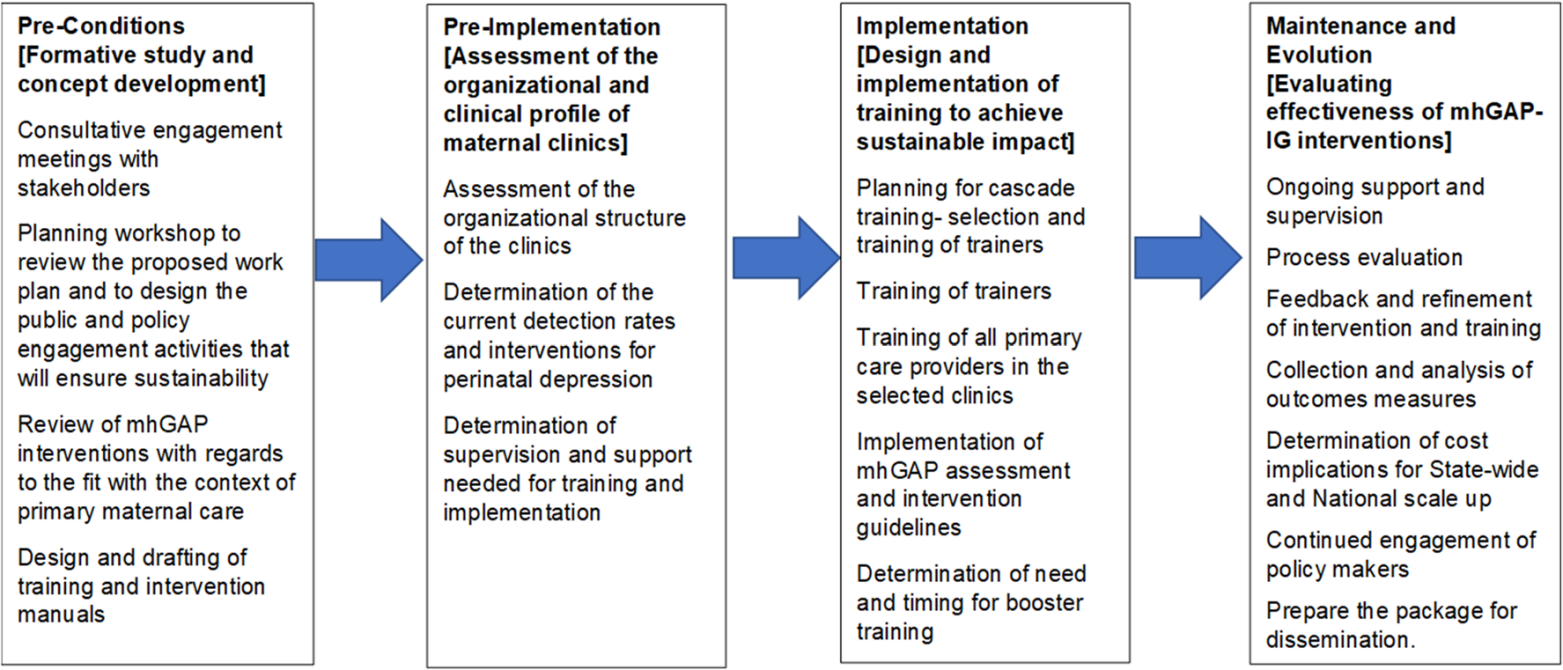
Replicating effective programs (REP) framework for the scaling up care for perinatal depression for improved maternal and infant health (SPECTRA) project

We shall be guided by the Replicating Effective Programmes (REP) framework as it provides a roadmap for maintaining treatment fidelity while providing opportunities to tailor intervention to fit local needs, as well as specifying training and technical assistance strategies to maximize the chances for sustaining the intervention [21]. Evidence based interventions for perinatal depression and programmes for implementation have been tested in a variety of settings. This study will explore the factors affecting the use of a cascade training format to build the capacity of frontline primary maternal care providers in the use of mhGAP-IG to deliver evidence-based intervention for perinatal depression. We will assess the effectiveness of the intervention delivered by the providers following training in the use of mhGAP-IG by comparing the outcomes of women with perinatal depression who present for care before with those who present after the training of the providers. In line with the REP model, the study will be carried out in four phases: 1. Pre-condition, 2. Pre-implementation, 3. Implementation and 4. Maintenance and evolution (Fig. 1).

This study will be implemented in the following four overlapping phases:

Phase 1 (Pre-Condition) (months 1–6): formative study and concept development.

Phase 2 (Pre-Implementation) (months 7–18): assessment of the organizational and clinical profile of maternal clinics.

Phase 3 (Implementation) (months 19–24): design and implementation of training to achieve sustainable impact.

Phase 4 (Maintenance and Evolution) (months 24–48): evaluating the effectiveness of mhGAP-IG intervention and evaluating gaps and bottlenecks in its delivery.

### Study setting

The study will be carried out in all the 11 local government areas (LGA) in and around the city of Ibadan. Patient recruitment will be from randomly selected primary health care clinics across the 11 LGAs. The selection will take into consideration the location of the clinics in either rural or urban setting. Each of the LGAs has an average of 10–14 primary health care clinics (PHC). From the average patient load of the clinics, we estimated that 20 clinics would be required to meet the calculated sample size within the study time frame (see below). Based on current estimated patient flow, the clinics were selected in such a manner that approximately half of the participants are recruited from rural and the other half from urban centres.

### Methods phase one

#### Engagement activities

We shall conduct consultative engagement meetings with stakeholder groups that include community leaders, policy makers, primary care providers and women to secure cooperation and obtain views relevant to the success of project implementation. Specifically, we shall conduct key informant interviews with selected facility managers involved in our previous trial to obtain information about how perinatal depression is viewed and understood and currently managed. Also, included in these engagement activities will be women who had perinatal depression at the time of our previous trial and had participated in it, and who had given permission to be contacted for future studies.

#### Planning workshop

A planning workshop will be organized, consisting of key players from our earlier mhGAP demonstration project and will include women who had received care for perinatal depression previously, primary health care workers (PHCW), nurses, primary care physicians, policy makers from the Ministry of Health as well as members of the research team. The focus of the workshop will be to: (1) review the programme of work relating to the project; (2) review experience of previous training of primary care workers using the mhGAP-IG and the mhGAP demonstration project and draw the relevant lessons from it; (3) design the public and policy engagement activities that will ensure project sustainability. A major outcome of the workshop will be the development of a Theory of Change map that highlights the assumptions, the barriers as well as the facilitators for successful project implementation and future scale-up [22].

### Methods phase two

#### Assessment of the organizational and clinical profile of maternal clinics

This will be conducted in two parts: (a) Review of facility care profile, and (b) Recruitment and assessment of Cohort One perinatal women.

#### Review of facility care profile

Depression is commonly a chronic disorder and should ideally be treated within a chronic care model. We therefore seek to address the question: what is the current care arrangement in this facility for chronic conditions? To address this question, we will conduct key informant interviews with the facility managers of the 20 selected clinics for the current study to enquire about process of care for perinatal women, the structural features of the clinics that may have a bearing on the care of women with perinatal depression and the administrative environment for training and supervision of the PHCW. These interviews will be supplemented by information collected using the Assessment of Chronic Illness Care (ACIC) [23], a tool specifically designed to obtain information about organizational structure of a health facility towards delivering effective intervention for chronic conditions. The tool has been previously adapted for LMIC settings and will be further adapted for the specific focus of this project.

### Recruitment and assessment of cohort one

We will recruit a cohort of women to address two questions during this phase of the study: What is the current rate of detection and treatment of perinatal depression? What is the experience of women receiving care in the clinics in regard to the attention given to their psychological health?

### Participants

Consecutively registered women presenting for antenatal care in the selected clinics will be screened using the Edinburgh Postnatal Depression Scale (EPDS) to identify women with depression [24]. Women with depression (scoring 10 or more on the EPDS), and who consent to further participation in the study will be recruited.

### Procedure

**Fig. 2:**
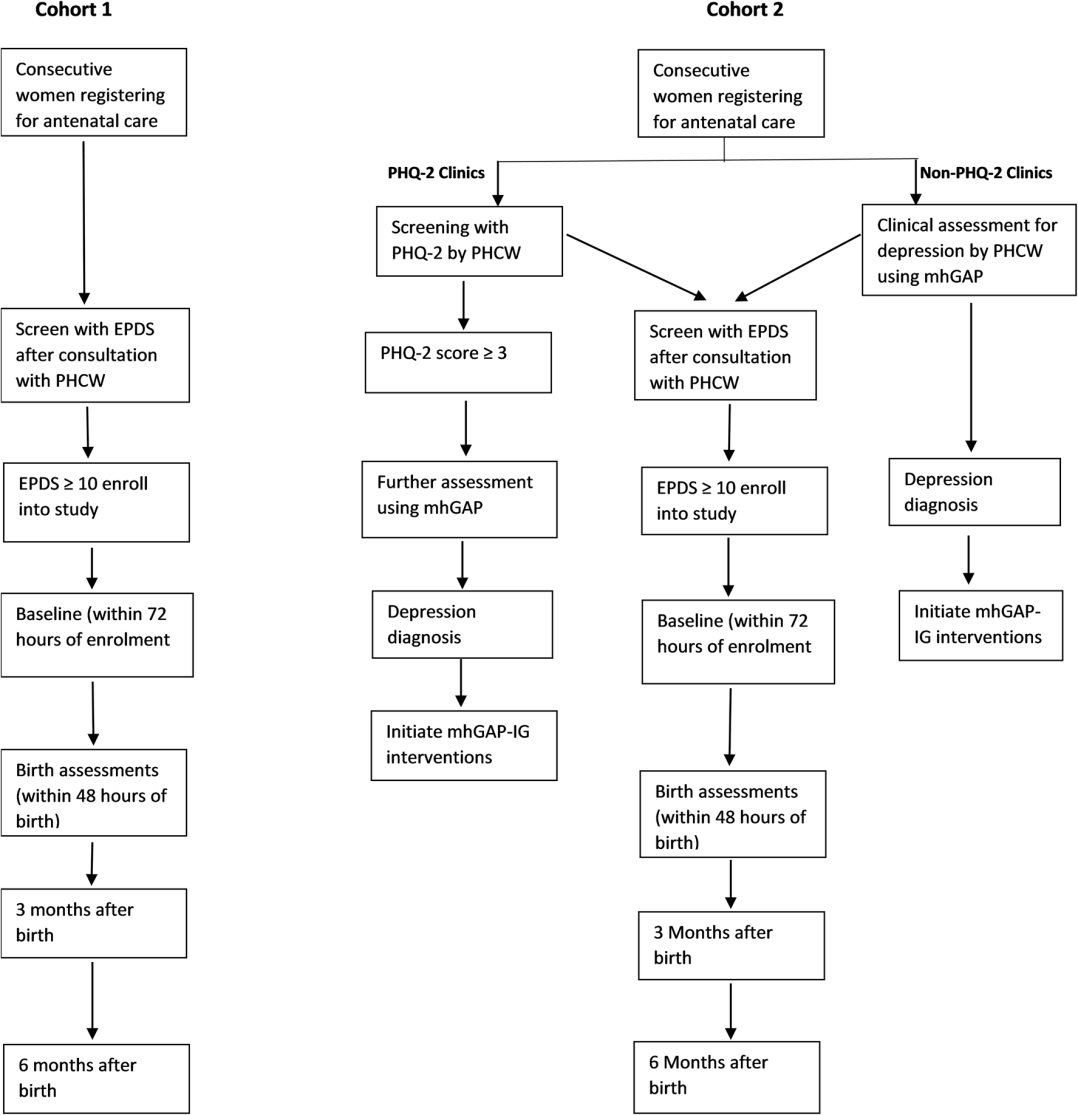
Flow of perinatal women in cohorts one and two

Consecutively registered women presenting for antenatal care will be screened with the EPDS after being attended to by the PHCW (Fig. 2 and SPIRIT guideline). All those who screen positive for depression, irrespective of whether they have been identified as having depression by the PHCW or not, and consent to participate in the study will constitute Cohort One. At an estimated prevalence of at least 10% and about 5% refusals, we expect to screen about 1800 women to recruit 167 women with scores at or above the cut-off level and consenting to participate in the study (see below for sample size estimation). This cohort will enable us determine how many of those who screen positive are identified by the PHCW and what treatment is offered to them. Even though this cohort is to document current treatment practices in these clinics for women with depression, any woman considered to be at high risk will be referred for treatment. This will include women with severe depression as indicated by an EPDS score of 18 or greater and those with suicidal ideation as indicated by a score of 3 or more on the tenth item of the EPDS. Severely depressed or suicidal subjects will be tracked to see if they took up referral and additional care, as part of process evaluation. Women who are too ill to cope with the interview or require urgent medical attention will be excluded from recruitment.

### Outcome assessments

Baseline assessments will be conducted by research assistants within 72 h of screening positive and consenting to enter into the study in the respondent’s home (or any other place of their choice) (Table 1). The primary outcome assessment will be at 6-month postpartum with secondary assessment points being at 3- and 12-month postpartum, all at the participant’s home. The outcome assessments from this cohort will be compared to those of the subjects in Cohort Two (see below) to determine the effectiveness of the interventions delivered following the training of the providers.

**Table 1:** Assessment timeline

Assessment time point	Cohort one	Cohort two
At registration for antenatal care	Assessed for eligibility	Assessed for eligibility
Enrollment	EPDS > 10 Informed consent	EPDS > 10 Informed consent PHCW institutes mhGAP interventions
Baseline (within 72 h of enrollment)	Demographic questionnaire Assessment of psychological care CSRI (SUQ) WHODAS PACIC	Demographic questionnaire Assessment of psychological care CSRI (SUQ) WHODAS PACIC
Birth	GA at birth Place of delivery Infant birth weight and head circumference	GA at birth Place of delivery Infant birth weight and head circumference
3 months after delivery	EPDS WHODAS CSRI Infant well-being assessment	EPDS WHODAS CSRI Infant well-being assessment
6 months after delivery	EPDS WHODAS CSRI Infant well-being assessment	EPDS WHODAS CSRI Infant well-being assessment
One year post-delivery	EPDS WHODAS CSRI Infant well-being assessment	EPDS WHODAS CSRI Infant well-being assessment

Respondents will be administered a battery of questionnaires including: a short questionnaire to enquire about whether they have been asked questions about their psychological health by the maternal care providers and their experience of care in the clinic, the Client Service Receipt Inventory-Postnatal version (CSRI-PND) to evaluate their use of service in the previous month [25], the World Health Organization Disability Assessment Scale (WHO-DAS) to determine their level of disability [26], and the Patient Assessment of Chronic Illness Care questionnaire [23] to further examine their assessment of the profile of care in the facility. These postpartum assessments will collect information about infant feeding, and the child’s vaccination history, experience of common illnesses (fever, diarrhoea) and developmental milestones.

### Methods phase three

#### Development of a training manual

We will develop a training manual for use by the Trainers with a guide that describes the methods of training primary care workers, including the organization of both didactic lectures, use of power point, and conduct of role plays.

### Cascade training

#### Participants

The supervisory physician and three of the most senior cadres of primary health care workers (this category include-nurses, senior community health officers [CHO]) from each of the 11 local government areas in which the study is to be implemented will be recruited as trainers (N = 44). These trainers will subsequently provide training for the end users- frontline primary care providers who deliver care for perinatal women. All the frontline providers in each of the selected clinics will be invited to participate in the trainings. We aim to train at least 200 frontline primary care workers.

### Procedures for cascade training

#### Training of trainers

**Fig. 3:**
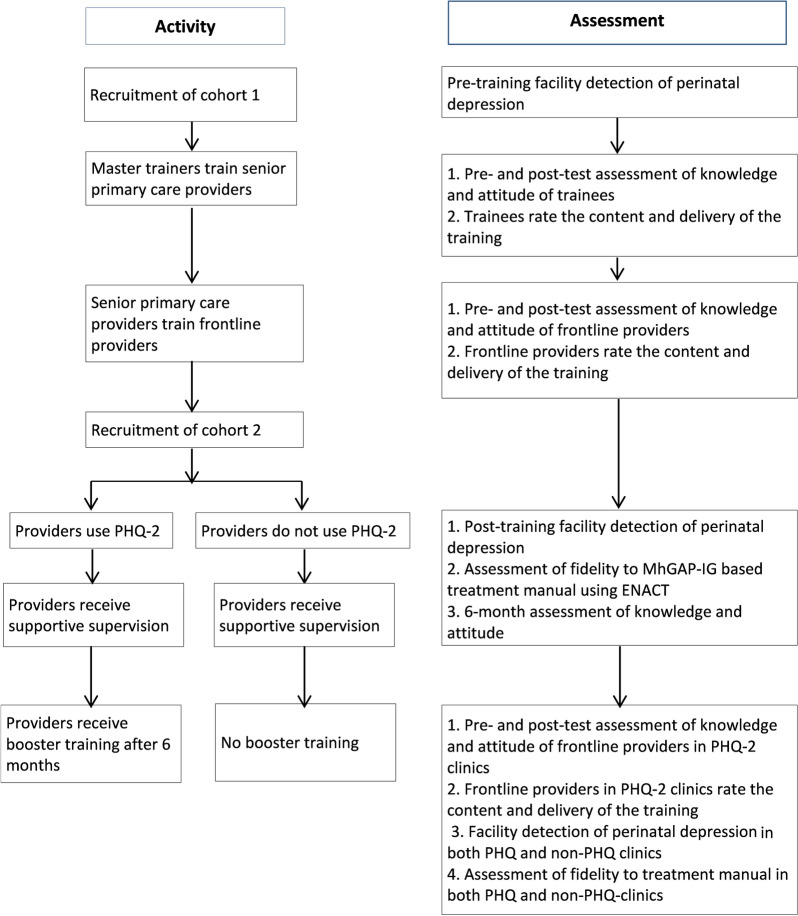
Cascade training model and assessments of trainers and providers

One Training of Trainers (ToT) workshop will be conducted by two psychiatrists (Master Trainers) with experience in the training of providers in the use of mhGAP-IG and perinatal mental health (Fig. 3). The trainers will be trained in the use of mhGAP-IG to manage perinatal depression (with a range of severity) and suicidality and how to train the end users and provide supervision and support to them.

#### Training of frontline primary care providers

Subsequent to the ToT, the trained trainers will conduct training workshops with the end-users of mhGAP-IG. This training will utilize the training materials developed by the Master Trainers and will be supervised by the Master Trainers. We plan for each training workshop to be facilitated by at least two of the trained Trainers. Each 2-day workshop will be attended by no more than 20 participants to ensure effective and interactive training. Participants will be tested pre- and post-training on knowledge and attitude. They will also provide structured ratings on the content and delivery of the training. At the end of each training workshop, the Master Trainer will have a de-briefing session with the Trainers during which a review of the workshop will be conducted and lessons learnt noted.

#### Use of a screening instrument

We will explore the impact of the incorporation of a short screening tool for depression into the routine clinical assessment of women presenting for antenatal care. After the training of the providers in the use of mhGAP-IG for the detection and treatment of perinatal depression, participating clinics will be divided into two groups. In one group of randomly selected clinics, providers will use the 2-item Patient Health Questionnaire (PHQ-2) to routinely screen consecutive women attending maternal clinics to determine need for further assessment for depression. Providers in the second group of clinics will not be using a screening tool before assessing the antenatal women for depression.

#### Assessing knowledge retention

Approximately 6 months after the initial training, 40 of the trained PHCW, randomly selected, will receive another test to determine the level of knowledge retention, the level of drift in the acquired skills and factors that are related to retention or drift. Such factors may be the characteristics of the providers, including their level of clinical experience, post-training performance, age, or level of clinical support.

#### Refresher training

Approximately 6 months after the initial training of frontline primary care providers, a refresher training essentially like the initial one will be provided for the PHCW in the PHQ-2-using clinics. Participants at this training will also be tested pre and post training.

#### Supportive supervision

The frontline providers in all the clinics will have structured fortnightly supportive supervision conducted by the trainers. A structured checklist will be designed to cover clinic organization and service delivery activities geared towards identifying and providing care for perinatal depression, on which the supervisors can score each clinic on a Likert scale. The supervisors will also provide technical and other assistance to the frontline providers as needed.

## Methods phase four

### Participants

Consecutive women making their first antenatal visit will be screened for depression with the EPDS after their consultation with the primary care providers (Fig. 2). Women who screen positive will be invited to participate in the study. At an estimated prevalence of at least 10% and about 5% refusals, we expect to screen about 3500 women to recruit 334 women with scores at or above the cut-off level (see section on sample size determination) and consenting to participate in the study. Women who screen positive, irrespective of whether they have been identified as depressed by the PHCW, and who provide informed consent to participate will constitute Cohort Two for the study. Those who screen positive but have not been identified by the primary care workers will be advised to see the providers again and notify them of their depression status. This will be particularly so for those who score 18 or more or have suicidal ideation on the EPDS and are therefore judged to have severe depression as well as those who endorse the item on suicidality on the EPDS. This is to ensure that they get timely treatment. The providers are also required to consult with the project psychiatrist for the management of patients with serious suicidal risk.

### Interventions

The interventions will be based on treatment specifications in the WHO Mental Health Gap Action Programme Intervention Guide (mhGAP-IG) as adapted for the health system of Nigeria [27]. The mhGAP-IG depression module provides detailed guideline for the management of moderate to severe depression with special consideration for pregnant or breastfeeding women. The mhGAP-IG emphasizes the use of psychosocial interventions for depression in pregnant and breast-feeding women with the lowest effective dose of an antidepressant being used when there is no response to psychosocial treatments. In line with this, interventions for perinatal depression in this study will include psychoeducation, addressing current psychosocial stressors and reactivating social networks.

Psychoeducation will be offered to every woman enrolled into the study as well as to their family members as appropriate. Psychoeducation involves an explanation of the diagnosis to the patient in simple language using local expressions while avoiding the labelling of ‘mental illness/disorder’. The patient is helped to understand that the symptoms being experienced are not as a result of laziness or supernatural forces but an ailment that is common and amenable to treatment. Addressing current psychosocial stressors entails offering the patient an opportunity to talk about current psychosocial problems and, to the extent possible, the health worker addressing pertinent social issues and assisting the patient to solve the problems with the support of available community resources. In reactivating social networks, the patient is encouraged to re-initiate prior social activities that have been neglected on account of the illness.

The health worker is expected to follow up the patient regularly. However, the choice of the number and frequency of the visits will be at the discretion of the attending health care provider. Low dose medications will be indicated for women who do not improve with the psychosocial treatments. Medication will only be given after consultation with the primary care physician.

## Outcome measures

### Primary outcomes

The primary implementation outcome is change in the identification of perinatal depression between the two cohorts by the providers. The primary effectiveness outcome is difference in remission rates from depression at 6 months between the two patient cohorts. Remission is defined as an EPDS score of 5 or less at 6 months post-partum.

#### Secondary effectiveness outcome

The secondary effectiveness outcome will be (1) the difference in the level of disability between the two patient cohorts at 6 months post-partum as assessed with the WHO-DAS [28]; and (2) difference in infant growth and development outcomes between the two cohorts.

#### Secondary implementation outcomes

A range of mixed-methods assessments will be conducted.

1. Assessment of quality of intervention: We will conduct a detailed assessment of fidelity with mhGAP-IG specifications for treating depression by the providers. This will be done by research supervisors sitting in during clinical encounters between PHCW and depressed women. We will use the 18-item Enhancing Assessment of Common Therapeutic factors (ENACT) rating scale [29] to evaluate the extent to which providers are using the skills acquired during the training to provide appropriate psychological assessment and intervention for the women. ENACT is designed to evaluate the delivery of psychological interventions, especially as prescribed in the mhGAP-IG. We will modify it to also include decisions about referral and use of medication. Twenty five consultations will be rated with the tool.
2. Assessment of contextual factors affecting delivery of intervention: Qualitative interviews will be conducted with selected PHCW (N = 20) and women who recover from depression and remain well through the follow-up period (N = 15) and those who fail to make consistent recovery (N = 15) to understand contextual factors that enable/inhibit the delivery of effective treatment using the mhGAP-IG. We will be interested to learn how integrated care of perinatal depression has improved overall health care (for example improved quality of communication with service users, improved service user satisfaction with care), how it has impacted on the functioning of the different components of care, including factors that facilitate or act as barriers to the delivery of intervention at a systems level (for instance information, human resources), as well as issues relating to stigma and discrimination.
3. Process evaluation: we will be conducting process evaluations alongside to enable us to (1) assess the quality of implementation, and (2) identify contextual factors that could affect the scaling up of interventions for perinatal depression. We will target two processes for detailed evaluation- the training of the primary care workers by the trained trainers and the delivery of the interventions for depression using the mhGAP-IG.
4. The training process: We will assess the changes in knowledge and attitude of the trainers following the ToT workshop using pre and post-test questionnaires, knowledge of depression scale [30], and depression attitude questionnaire [31] administered before and after the training sessions. We will evaluate the extent to which trainers demonstrate fidelity to the training procedure when they train the PHCW. Master trainers will sit in at the training workshops delivered by the trained trainers as non-participant observers. During these observations, the master trainers will document their assessment of the training procedure using a semi-structured observation pro forma specifically designed for this purpose. Some of the cascade training sessions will be video-recorded for further training purposes.
5. The Trainees: We will similarly assess the changes in the knowledge and attitude of the trainees who will be at the frontline of delivering the interventions. This will be done using: (1) Pre- and post-test questionnaires, (2) Selected items from the Knowledge of depression scale, (3) Depression attitude questionnaire (4) Satisfaction with training- a semi-structured questionnaire designed to capture: what they liked most about the training, what they had not understood about training topics, what they would do differently as a result of the training, (5) Changes in practice after the training 6) Retention of knowledge and changes in attitude- (at 3, 6, 12 months after training) using Post-test questionnaire, Knowledge of depression scale and the Depression attitude questionnaire.
6. Other process evaluation activities: We will document and link to our Theory of Change Map findings of assumptions/potential barriers and facilitators. We will also track and monitor turnover, availability and transfer of mhGAP-trained staff and supervisors, frequency of supervision sessions, women's attendance for follow-up sessions, and also track any contextual changes in the course of the study.

4. Observational component: Throughout the study period, trained research assistants and field supervisors will pay both scheduled and unscheduled visits to the facilities to document both the visible existing organisational and operational aspects of delivering care for perinatal depression using a pre-designed pro forma. This is essentially to supplement and triangulate the information obtained from key informant interviews and focus group discussions.

5. Effects of COVID-19: Although unexpected, the pandemic might affect the later stages of the recruitment and follow up period. If this happens, we plan to track factors related to COVID-19, for example, in terms of patient flow, ease and regularity of follow up, any increase in domestic violence due to lock down, given its strong association with depression in women.

### Blinding and protection against sources of bias

The following steps will be taken to reduce the risk of bias in this study: (1) The study is designed to ensure that the risk of contamination between clinic clusters is low as women are unlikely to move from clinics using PHQ-2 and those that are not, due to their geographical spread and because there will be no publicity regarding the use of PHQ-2 in some clinics. (2) Outcome assessors at the women’ homes are not involved in screening women at the clinics, and are blind to whether women received care in PHQ-2 clinics or not.

### Data collection and quality control

Quality control of field work will be implemented by research supervisors and this includes random checks on the quality of interviews (conducted by physically observing at least 10% of the interviews conducted by a research assistant). Supervisors will also work with the Data Manager to check that research assistants have correctly captured study data.

### Data protection

All data will be kept anonymously by using codes to identify individuals. Data will be uploaded to a server located in the central office where it will be cleaned and stored. Access to the datasets is possible for members of the research team through a password-protected entry.

### Sample size

Experience from the control arm of our recently concluded randomized controlled trial of intervention for perinatal depression suggests that just over 70% recover at 6 months [32]. The control arm of that study received low intensity intervention based on the specifications of mhGAP-IG and delivered by trained PHCW. We will expect about the same rate of recovery in the current study. Prior to training, we assume a recovery rate of 55% at 6 months following delivery based on previous observations by us [19]. We think that this difference of 15% is clinically meaningful to promote changes in routine maternal care. In the said RCT, we were able to complete primary outcome data at 6 months postnatal for about 85% of the participants. We will plan to recruit about twice as many mothers after the training of the providers than before the training (that is a ratio of 2:1 following training) in order to provide more information about contextual and health system factors that help or hinder effective implementation. We estimate a sample of 167 prior to training and 334 post-training to detect a difference of 70% vs 55% (equivalent odds ratio = 1.8) with 80% power at the two-sided 5% alpha level.

The correlations between the successive pre- and post-training tests conducted for the providers during the demonstration project in the State of Osun was 0.37 and the within subject variance was 0.352 [18]. Using these values and with a projected power of 80% and Type I error of 0.05, we estimate the number required to detect a difference of 1 unit between tests scores (considered a meaningful change in the assessment tool we plan to use) to be 112. Assuming an attrition of 15%, we will need a total of 129 providers to demonstrate a meaningful change between pre- and post-training scores as well as stability of skills during refresher training on average 3 months after the first training.

### Study instruments

*Assessment of Chronic Illness Care (ACIC)* [23] a tool specifically designed to obtain information about organizational structure of health facility towards delivering effective intervention for chronic conditions. The tool has been previously adapted for LMIC settings and will be further adapted for the specific focus of this project. This tool will be administered to facility managers by trained research assistants during the formative phase of the project.

*Patient Assessment of Chronic Illness Care questionnaire (PACIC)* [23] will be used to evaluate patient’s perspective on receipt of care delivered in the facility. This instrument will be administered to participating antenatal women in both cohorts during the follow up periods.

*Edinburgh Post Natal Depression Scale (EPDS)*: The EPDS is a 10-item screening instrument for depression. It has been validated and used in earlier studies of perinatal depression in Nigeria [33]. The EPDS will be used by trained research assistants to screen consecutive women presenting for their first antenatal visit after their consultation with the primary care providers, to determine their eligibility for enrolment into the study.

*Patient Health Questionnaire-2 (PHQ-2)* [34]: This is a two item screening instrument for rapid screening of depression. It consists of the first two items of the 9-item Patient Health Questionnaire (PHQ-9) [35], which has been validated among Nigerian students [36]. This tool will be used by previously trained primary care providers in half of the participating maternal clinics to assist them in the detection of perinatal depression.

*Client Service Receipt Inventory-Postnatal version* (CSRI-PND) will be used to evaluate the participating mothers’ use of service in the month prior to the assessment time point [25]. The CSRI-PND is an adaptation of the Service Utilization Questionnaire (SUQ) [37] which we have used in previous studies in collaboration with its principal designer. We shall use this instrument in follow-up assessments by research assistants, to systematically collect resource-use data of the participating perinatal women, including any inpatient care, consultations with health providers, use of drugs and laboratory tests, and also time and travel costs associated with this service uptake. We have recently adapted the CSRI-PND for the Nigerian health situation by applying it to antenatal women [19].

*WHO Disability Assessment Schedule (WHO-DAS):* The WHODAS was developed for measuring functioning and disability in accordance with the International Classification of Functioning, Disability and Health across different populations. The WHODAS II has high internal consistency (Cronbach’s alpha, α: 0.86), a stable factor structure; high test–retest reliability (intra-class correlation coefficient: 0.98); good concurrent validity in patient classification [28]. The WHO-DAS will be used by trained research assistants to collect disability data of the participating perinatal women during the follow up period.

*Infant well-being assessments*: the current level of cognitive, language, personal-social, and fine and gross motor development of the offspring of the perinatal women will be assessed by research assistants with an infant well-being questionnaire. This questionnaire was developed by us and it incorporates questions on child nutrition, achievement of developmental milestones, illnesses, immunization and measurements of the length/height and weight and administered at 3 months, 6 months and one year post delivery.

*Enhancing Assessment of Common Therapeutic factors (ENACT)* rating scale: This is an 18-item tool that has been developed to provide reliable and valid assessment of therapist competence in a variety of cultural and service settings [29]. The tool will be used by research supervisors to evaluate the fidelity of the care providers during clinical encounters between them and the depressed women.

*Knowledge of Depression Scale*: This is a 27-item multiple choice questionnaire designed to assess basic knowledge about depressions and its treatments [30]. Selected items from the tool will be used to test change in knowledge of frontline service providers during the training process as well as to assess knowledge drift in the periods after the training.

*Revised depression attitude questionnaire (R-DAQ)* is a 22-item tool for examining clinicians’ views and understanding of depression [31]. Item from the R-DAQ will be administered to frontline providers to assess changes in attitude during and after their training workshops.

### Data analysis

Qualitative interviews will be transcribed. Interviews conducted with women will be conducted in Yoruba and will be translated into English following which back-translation checks will be applied. The data generated will be analysed using thematic analysis with the assistance of a qualitative software package such as MAXQDA. We will use descriptive statistics to assess balance between Cohorts One and Two at baseline for both PHC and individual participant characteristics. In order to take appropriate account of the hierarchical nature of the data, we will use multivariate mixed effects logistic regression to estimate recovery from depression at three and 6 months for Cohort Two versus Cohort One, adjusting for baseline depression. These analyses will be repeated for depression, disability, and service use characteristics of the mothers and the growth profiles of the infants. We will conduct sensitivity analyses to assess the potential effect of missing data using multiple imputation methods. We will investigate the effect of adherence with the intervention using instrumental variable regression. Appropriate interaction terms will be entered into the primary regression analyses for recovery from depression in order to conduct pre-specified subgroup analyses that will include baseline symptom severity (EPDS score 10–18 vs 18 +) and duration (≤ 1 month¸ > 1 month). Data from the ACIC and the PACIC will be analysed using descriptive statistics. Quantitative data from the patient flow observation checklist will be analysed using descriptive statistics. All analyses will be conducted using STATA.

### Study status

**Fig. 4:**
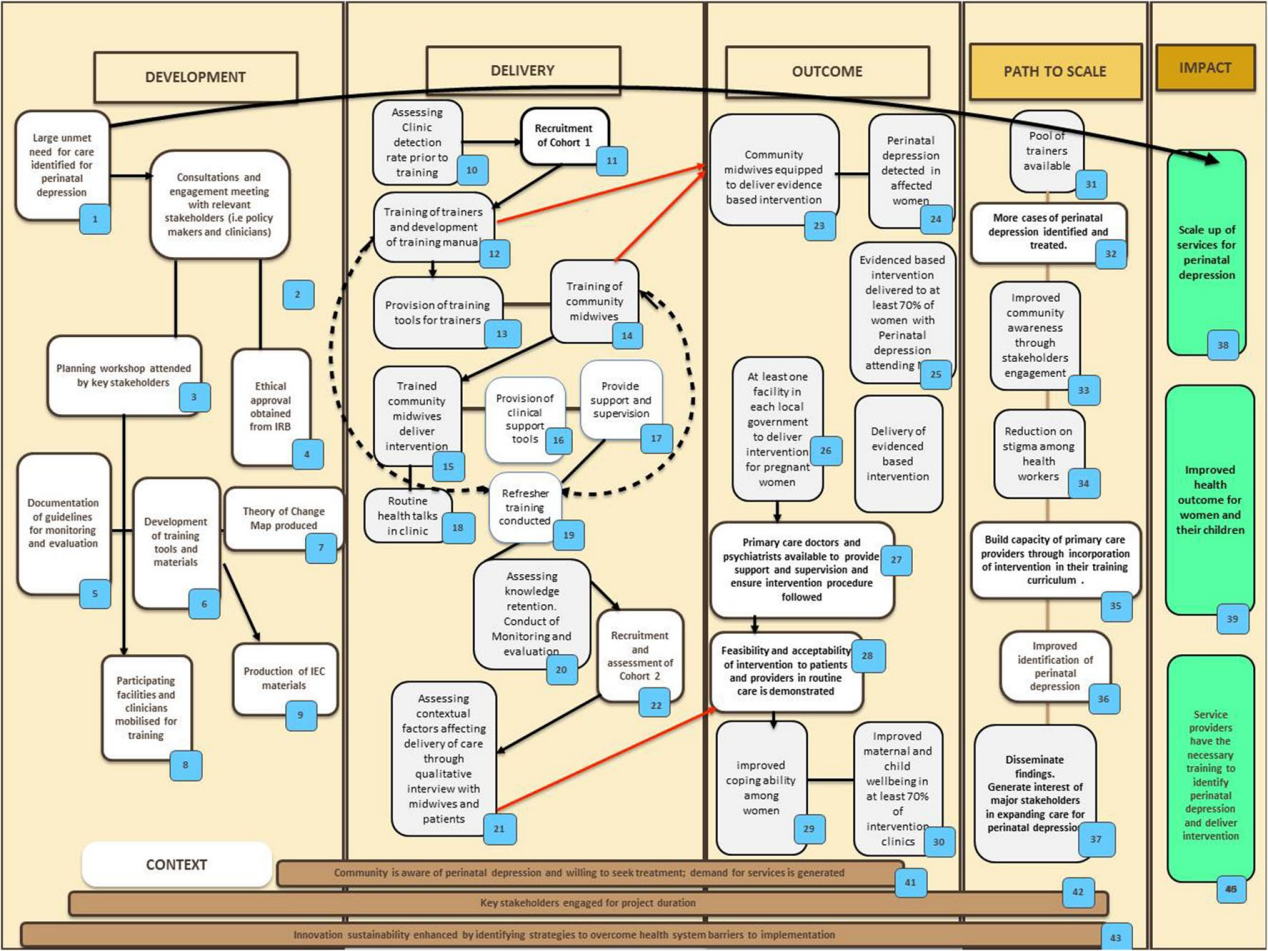
Theory of change map for the scaling up care for perinatal depression for improved maternal and infant health (SPECTRA) project

At the time of submitting this manuscript, the SPECTRA study is in the implementation phase. A theory of change map was produced from the formative phase of the study (Fig. 4). The recruitment of the first cohort of participants and six-month post-delivery assessments have been completed. The training of trainers’ workshops was carried out. The trained trainers have completed the stepped-down cascade training of other health care workers in all the selected clinics. The recruitment of the second cohort of women as well as the 6-month postnatal assessments have been done for all participants. One year post-delivery assessment is currently ongoing. Experience on the field as the study progressed led to several iterations of the study protocol, each of which required ethical approval. This hampered the decision to publish until the final version of the work plan was clear and ethical approval was obtained.

## Discussion

This study represents an attempt to systematically assess and document an implementation strategy that could inform the scaling up of evidence based interventions for perinatal depression using the WHO mhGAP intervention guide in low and middle income countries. While effectiveness trials have demonstrated that evidence based interventions for perinatal mental disorders can be delivered by trained primary care workers, there is need to understand contextual and other factors that may facilitate or impede the scaling up of these interventions in routine maternal care. Considering the prevailing resource constraints that characterize the mental health care systems across most LMICs, pragmatic and innovative approaches of providing training, support and supervision to primary care workers to improve the delivery of mental health care are needed. A feasible and sustainable approach is to equip more senior and experienced primary care providers with the necessary skills required to train and supervise the frontline health care workers in the delivery of care for common mental disorders with mental health specialists providing support.

In the formative phase of SPECTRA, a key issue that was raised by stakeholders is how to improve the identification of perinatal depression by the trained providers. While training primary care providers is known to improve identification, available evidence suggests that, even in settings with high resources, up to 50% of women with the condition are not correctly identified. Studies have shown that the use of a depression screening instrument could aid the identification of such women [38]. In our previous trials of the effectiveness of depression treatment, we have used trained research staff to administer screening instruments to identify participants with probable depression for primary care providers to assess and make the diagnosis of depression [19, 39]. We are yet to explore the feasibility of primary care providers incorporating the use of a depression screening tool into routine patient assessment. To assess this, at the implementation phase, half of the selected clinics will be randomly assigned to routinely administer a brief depression screening tool, the 2-item Patient Health Questionnaire (PHQ-2), to all women as a part of their assessments at each antenatal and postnatal visit. Women scoring 3 or more will be further assessed using the mhGAP-IG. In addition to improving the recognition of depression, routine screening might have the potential to enable health care providers to target women who are more at risk for further evaluation and thereby saving time. Our study will provide useful information about the feasibility, acceptability and impact of this approach in the identification and treatment of perinatal depression in routine maternal care in resource-constrained settings.

We acknowledge the potential limitations of our study design. For example, in a classic uncontrolled before-and-after study design like ours, changes observed in the clinics in terms of detection and treatment of perinatal depression, post intervention, may not be due to our intervention alone. They may also be due to other interventions not anticipated by us, such as new and unforeseen in-service training exposure for frontline primary care clinicians outside of our programme. We do not anticipate this in the life of the project because of the rarity of such opportunities to the clinicians. To take account of such unlikely development, approaches that are less prone to confounding of this sort, such as controlled before-and-after study design or a stepped wedge design might be suitable. We also note the limitation of self-report measures which may be prone to desirability tendency. However, we are collecting every information anonymously and clinicians are aware that no information provided will be used or available for assessing their job performance.

## Data Availability

The datasets generated and/or analysed during the current study are not publicly available due to local ethical committee restrictions but are available from the corresponding author on reasonable request.

## Abbreviations

ACIC: Assessment of chronic illness care
CHW: Community health worker
CHO: Community health worker
CHEW: Community health extension worker
CSRI-PND: Client service receipt inventory-postnatal version
EPDS: Edinburgh postnatal depression scale
ENACT: Enhancing assessment of common therapeutic factors
HIC: High income countries
LGA: Local government area
LMIC: Low and middle income countries
MCC: Maternal care clinics
MCH: Maternal and child health
MhGAP-IG: Mental Health Gap Action Programme Intervention Guide
PACIC: Patient Assessment of Chronic Illness Care questionnaire
PHC: Primary health care
R-DAQ: Revised depression attitude questionnaire
REP: Replicating effective programmes
PHCW: Primary health care worker
SPECTRA: Scaling up care for perinatal depression for improved maternal and infant health
SUQ: Service utilization questionnaire
ToT: Training of trainers
WHO-DAS: World Health Organization Disability Assessment Scale
